# Circulating FABP-4 Levels in Patients with Atherosclerosis or Coronary Artery Disease: A Comprehensive Systematic Review and Meta-Analysis

**DOI:** 10.1155/2023/1092263

**Published:** 2023-11-17

**Authors:** Narges Jalilian, Reza Pakzad, Mahdi Shahbazi, Seyyed-Reza Edrisi, Karimeh Haghani, Mohsen Jalilian, Salar Bakhtiyari

**Affiliations:** ^1^Department of Medical Biochemistry, Faculty of Medical Sciences, Ilam University of Medical Sciences, Ilam, Iran; ^2^Student Research Committee, Ilam University of Medical Sciences, Ilam, Iran; ^3^Department of Epidemiology, Faculty of Health, Ilam University of Medical Sciences, Ilam, Iran; ^4^Health and Environment Research Center, Ilam University of Medical Sciences, Ilam, Iran; ^5^Department of Health Education and Promotion, Faculty of Health, Ilam University of Medical Sciences, Ilam, Iran; ^6^Feinberg Cardiovascular and Renal Research Institute, Northwestern University, Chicago, IL, USA

## Abstract

**Background:**

Cardiovascular diseases (CDs), notably coronary artery disease (CAD) due to atherosclerosis, impose substantial global health and economic burdens. Fatty acid-binding proteins (FABPs), including FABP-4, have been recently linked to CDs. This study conducted a systematic review and meta-analysis to examine FABP-4 levels in CAD and atherosclerosis patients, exploring their potential links to these conditions.

**Methods:**

A systematic review and meta-analysis were done based on the PRISMA guideline. The international databases including Medline, Embase, Cochrane Library, Scopus, Web of Science, and UpToDate were searched to find all related studies on the effect of FABP-4 on patients with CAD or atherosclerosis which were published till June 2022 without language restriction. The Cochran's *Q*-test and *I*^2^ statistic were applied to assess heterogeneity, a random effect model was used to estimate the pooled standardized mean difference (SMD), a metaregression method was utilized to investigate the factors affecting heterogeneity between studies, and Egger's test was used to assess the publication bias.

**Results:**

Of 1051 studies, 9 studies with a sample size of 2327 were included in the systematic review and meta-analysis. The level of circulating FABP-4 in the patient groups was significantly higher than in the control groups (SMD = 0.60 (95% CI: 0.30 to 0.91, *I*^2^: 91.47%)). The SMD in female and male patients were 0.26 (95% CI: 0.01 to 0.52, *I*^2^: 0%) and 0.22 (95% CI: 0.08 to 0.35, *I*^2^: 44.7%), respectively. There was considerable heterogeneity between the studies. The countries had a positive relationship with heterogeneity (coefficient = 0.29, *p* < 0.001); but BMI, lipid indices, gender, study design, and type of kit had no effect on the heterogeneity. No publication bias was observed (*p*: 0.137).

**Conclusion:**

In summary, this meta-analysis revealed elevated circulating FABP-4 levels in CDs, suggesting its potential as a biomarker for these conditions. Further research is warranted to explore its clinical relevance.

## 1. Introduction

Cardiovascular diseases (CDs), notably coronary artery disease (CAD), impose significant clinical and economic burdens. CAD, primarily driven by atherosclerosis, stands out among the major CDs due to its substantial impact. This multifaceted disease is influenced by various physiological mechanisms. Atherosclerosis, characterized by cholesterol-laden plaque buildup in coronary arteries, is a central player. Endothelial dysfunction, inflammation, and oxidative stress contribute to plaque formation and instability. Elevated LDL cholesterol levels promote plaque development, while thrombosis can result from plaque rupture, leading to blood clot formation and artery blockage. Additional risk factors include hypertension, diabetes, genetic predisposition, and unhealthy lifestyle choices like smoking and poor diet [1, 2].

Obesity is a well-established risk factor for CAD [3]. Adipose tissue, functioning as an endocrine gland, releases adipokines [4]. These adipokines play crucial roles in lipid and glucose metabolism, as well as insulin sensitivity [5]. Past studies have shown that adipokines increase the risk of metabolic syndrome and CDs [5, 6]. One group of adipokines secreted from adipose tissue is the fatty acid-binding proteins (FABPs), which are a family of nine low molecular weight proteins (14 kDa-15 kDa) with tissue-dependent expression patterns. FABPs act as cytoplasmic lipid chaperones and function in the cellular transport of fatty acids [7].

Adipose fatty acid-binding protein (A-FABP) binds intracellularly to proteins [5] and regulates lipid metabolism and inflammation [7]. In addition to being expressed in mature adipocytes, this protein, also known by other names such as FABP-4 or aP2, is also expressed in macrophages [8] and generally constitutes about 6% of soluble proteins in adipose tissue [9].

A direct relationship has been shown between FABP-4, diabetes, and atherosclerosis in mice [9]. Some studies have shown that FABP-4 can predict the development of metabolic syndrome (MetS) [10] and type 2 diabetes mellitus [11, 12]. Other studies have shown that in mice with apolipoprotein E deficiency, FABP-4 gene deficiency protects these mice from atherosclerosis [9]; therefore, FABP-4 inhibition effectively treats atherosclerosis in a mouse model [13]. Further, a study in China has shown that there is an independent relationship between FABP-4 serum levels and carotid atherosclerosis in women [13]. It has also been shown that high FABP-4 serum levels can be related to the prediction and diagnosis of obesity-related metabolic syndrome [7]. In confirmation of the above studies, Furuhashi et al.'s study showed that a small molecule inhibitor of FABP-4 is an effective treatment option for severe atherosclerosis and type 2 diabetes in a mouse model [13]. On the other hand, Jin et al.'s study showed a significant relationship between the FABP-4 level and CAD only in women, while in this study, this relationship was insignificant for men with CAD [9].

In general, in recent years, several studies on the relationship between FABP-4, atherosclerosis, and CAD, though the results were not consistent, have been conducted. As the population of these studies was not large enough, their results cannot be generalized. Therefore, to better evaluate the relationship between circulating FABP-4 levels and CDs, more studies are needed. This study is aimed at investigating the relationship between circulating FABP-4 and CDs using a systematic review and meta-analysis. To the best knowledge of the researchers, this study is the first one that probes the relationship between circulating FABP-4 and patients with CDs. Provided that a significant relationship between FABP-4 levels and CDs is proven, the results of this study can be used to help diagnose and prevent CDs.

## 2. Materials and Methods

### 2.1. General Information

All the study procedures were reported based on the Preferred Reporting Items for Systematic Reviews and Meta-Analysis (PRISMA) guidelines [14] with CRD42022306267 registration number in PROSPERO.

### 2.2. Search Strategy

We conducted a comprehensive systematic literature review of online databases, including Medline, Embase, Cochrane Library, Scopus, Web of Science, and UpToDate. All analytical-observational studies such as case-control, cross-sectional, and cohort studies were included without any restrictions on the language of the articles. The search for data was done until June 2022. Studies assessed FABP-4 levels in both the case (patients with atherosclerosis or coronary artery disease) and control (noncardiovascular patients) groups which were selected for follow-up analyses. The search was conducted using strings including “coronary artery disease”, “coronary Arteriosclerosis”, “coronary Atherosclerosis”, “atherosclerosis”, “FABP-4 Protein, human”, “Adipocyte lipid-binding protein, human”, “fatty acid Binding Protein 4, Human”, “serum”, and “plasma”. The PICO in this study is as follows:

Population: patients with atherosclerosis or coronary artery disease

Intervention/exposure: circulating level of FABP-4 (human) in cardiovascular patients

Comparison: circulating level of FABP-4 (human) in noncardiovascular patients

Outcome: cardiovascular disease

Table 1 summarizes the process of the search strategy. The search was initiated in Medline (PubMed interface) and then was done in other databases. Google Scholar was used to access grey literature [15]. A biochemist expert was consulted for access to special and important articles.

### 2.3. Inclusion and Exclusion Criteria

All the case-control, cross-sectional, and cohort studies that examined the level of circulating FABP-4 in the patient group (patients with atherosclerosis or coronary artery disease) compared with the control (noncardiovascular patients) group were included in this study. Once the relevant studies were obtained, they were checked for duplicates using EndNote X6. Then, two rounds of screening were conducted. First, the titles and abstracts of the studies were checked. Second, the full texts of the articles were reviewed. The two steps were independently carried out by two authors of the study (N.J and M.SH), and any disagreement was resolved through consulting a third author (S.B). It should be mentioned that the kappa coefficient between the two rates was 93%. Blinding and task separation were done in the study selection procedure.

### 2.4. Data Extraction

Besides the name of the authors, country, date of publication, design of the study, sample size, gender, age, and kit type, other information such as mean; standard deviation (SD) of the circulating level of FABP-4 in the cardiovascular and noncardiovascular groups; lipid profile including low-density lipoprotein (LDL), high-density lipoprotein (HDL), total cholesterol (TC), and triglycerides (TG); and body mass index (BMI) in both groups was extracted.

### 2.5. Subgroup Definition

We run subgroup analyses based on gender, design of studies, country, and kit type for assessing the circulating level of FABP-4 in the case and control groups.

### 2.6. Risk of Bias

The Newcastle-Ottawa Scale was applied to evaluate the quality of selected studies [16, 17]. Two authors reviewed the articles separately, and then, the total score of each article was calculated.

### 2.7. Statistical Analyses

We performed the analyses using Stata software 11 (College Station, Texas). For each study, the mean value and SD of the circulating level of FABP-4 in cardiovascular and noncardiovascular were extracted, and when the IQR was reported, we changed it to SD with IQR/1.35 [18]. It should be noted that if FABP-4 had different measurement units in primary studies, all units are converted to Ng/ml. Then, the standardized mean difference (SMD) of the circulating level of FABP-4 in cardiovascular and noncardiovascular for each study was calculated based on Cohen's *d* formula as follows:

(1) $$\begin{array}{c} {\text{Cohen}}^{\prime }\text{s SMD}=\frac{M_{1}-M_{2}}{{\text{SD}}_{\text{pooled}}},\\ {\text{SD}}_{\text{pooled}}=\sqrt{\frac{\left(n_{1}-1\right)\text{S}{\text{D}}_{1}^{2}+\left(n_{2}-1\right)\text{S}{\text{D}}_{2}^{2}}{n_{1}+n_{2}-2}}, \end{array}$$

where *M*_1_ is the mean values of groups with CDs, *n*_1_ is the sample size of groups with CDs, SD_1_ is the SD of groups with CDs, *M*_2_ is the mean values of groups without CDs, *n*_2_ is the sample size of groups without CDs, and SD_2_ is the SD of groups without CDs. Then, the calculation of pooled SMD was done by the “Metan” command [19]. Positive or negative SMD means a higher or lower value of FABP-4 in the CD groups compared to the control group. Cochran's *Q*-test of heterogeneity was applied to determine the heterogeneity, and the *I*^2^ index was used as an indicator to quantify heterogeneity. In accordance with Higgins' classification approach, *I*^2^ values above 0.7 were considered high heterogeneity. To estimate the pooled SMD for FABP-4 and subgroup analyses (based on gender, kit type, country, and study design), the fixed-effect model was used, and when the heterogeneity was greater than 0.7, the random effect model was used. The metaregression analysis for examining the effect of kit type, study design, country, quality score, BMI, gender, and lipid profile (HDL, LDL, TC, and TG) as factors affecting heterogeneity among studies was run. The check for publication bias was conducted using the “Metabias” command, and in any case of possible bias, the pooled SMD was adjusted with the “Metatrim” command using the trim-and-fill method. In all analyses, the significance level was set at <0.05.

## 3. Results

**Figure 1:**
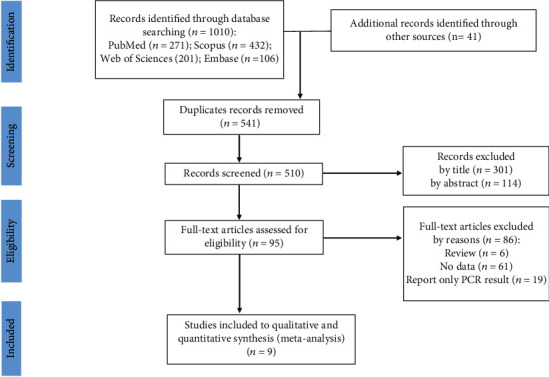
PRISMA 2020 flow diagram for new systematic reviews which included searches of databases and other sources.

A total of 1051 studies were retrieved from all databases and sources. After screening the studies for duplicates, 541 studies were eliminated, and 510 studies were remained. After applying the eligibility criteria, 415 studies were excluded by title and abstract, and 95 studies were remained. In the next step, 86 studies were excluded by full-text screening, and finally, 9 studies with a 2327 total sample size (1332 in the case group and 995 in the control group) were included in the systematic review and meta-analysis [5, 7, 9, 11, 20–24]. Five studies evaluated CAD, and 4 studies evaluated atherosclerosis. The characteristic of the included studies is shown in Table 2, and the selection process is shown in Figure 1. Japan and China had the highest numbers of studies (Japan = 3, China = 3). All nine studies were published between 2009 and 2020. Rhee et al. [7] (mean age = 60.7 ± 10 years old) and Kajiya et al.'s [5] (mean age = 69 ± 11 years old) studies had the lowest and highest age mean, respectively. Four studies were case-control, and five studies had a cross-sectional design. The results of the quality assessment are shown in supplement Tables 1 and 2.

### 3.1. Mean Difference Pooled Estimate of FABP-4

**Figure 2:**
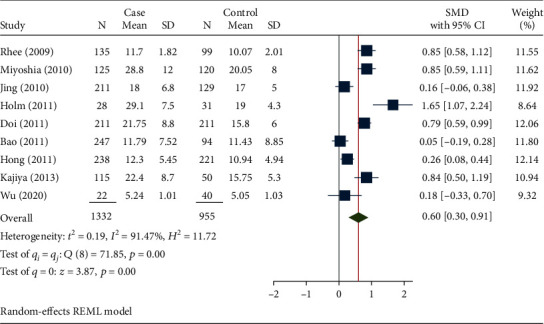
Forest plot for the pooled standardized mean difference of FABP-4 based on a random effect model. Each study identifies the first author (year). Each line segment's midpoint shows the standardized mean difference, the length of the line segment indicates 95% CI in each study, and the diamond mark illustrates the pooled estimate.

Figure 2 shows a forest plot of the standardized mean difference of circulating FABP-4 between patients with CDs and without CDs. Bao et al. [11] reported the minimum standardized mean difference of FABP-4 (0.05; 95% CI: -0.19 to 0.28) in China, and Holm et al. [21] showed the highest SMD of FABP-4 in Norway (1.65; 95% CI: 1.07 to 2.24). Based on Figure 2, the pooled estimate of the SMD was 0.60 (95% CI: 0.30 to 0.91, *I*^2^: 91.47%). Therefore, compared to healthy individuals, the mean of FABP-4 was significantly higher in individuals with CD.

### 3.2. Pooled Standardized Mean Difference Based on Different Subgroups

**Figure 3:**
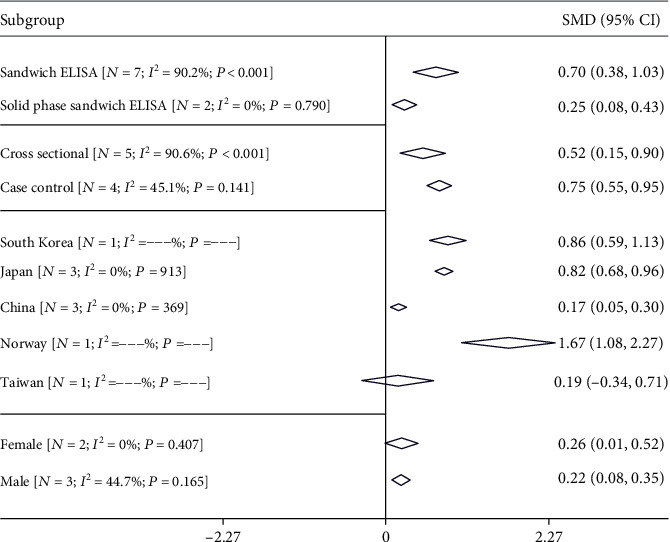
Pooled estimate with 95% CI and heterogeneity indices of the standardized mean difference of FABP-4 based on the type of the kit, country, and study design. The diamond mark illustrates the pooled estimate, and the length of the diamond indicates the 95% CI. *N* is the number of the study in each subgroup.

Figure 3 shows the results of the pooled SMD for the four subgroups. As shown, pooled SMD in the sandwich ELISA group was 0.70 (95% CI: 0.38 to 1.03, *I*^2^: 90.2%) and 0.25 for the solid phase sandwich ELISA group (95% CI: 0.08 to 0.43, *I*^2^: 0%). Also, based on the study design, the pooled SMD in the cross-sectional study was 0.52 (95% CI: 0.15 to 0.90, *I*^2^: 90.6%) and in the case-control studies was 0.77 (95% CI: 0.55 to 0.95, *I*^2^: 45.1%). Furthermore, the pooled SMD in females was 0.26 (95% CI: 0.01 to 0.52, *I*^2^: 0%) and in males was 0.22 (95% CI: 0.08 to 0.35, *I*^2^: 44.7%). The pooled SMD for a different country is summarized in Figure 3.

### 3.3. Heterogeneity and Metaregression

There was significant heterogeneity between the studies (*p* < 0.001). The *I*^2^ index for the total SMD was up to 90% (Figure 2). The result of metaregression is shown in supplement Table 3. Metaregression results showed that the country variable (coefficient: 0.29, *p* < 0.001) had a significant effect on the heterogeneity of the studies. It means that, on average, the SMD of FABP-4 in Taiwan was 0.29 more than that of China, or in Japan more than that of Taiwan. HDL (Supplement Figure 1A), LDL (Supplement Figure 1B), and other variables including kit type, study design, quality score, gender, BMI, TG, and TC shown in supplement Table 3 had no effect on the heterogeneity of the studies.

### 3.4. Publication Bias

Egger's test revealed no significant publication bias in the present meta-analysis (*Z* score: 1.49, *p*: 0.137) (Supplement Figure 2).

## 4. Discussion

FABP-4 is an emerging adipokine that plays an important role in the development of metabolic syndrome and type 2 diabetes as well as complications resulting from these disorders, including atherosclerosis [13, 24]. The relationship between this protein and the severity of atherosclerosis has been confirmed in several studies [5, 7, 13, 22]. In this research, the number of included studies was suitable for meta-analysis; moreover, since some studies encompassed subgroups, the combined effect size was also calculated. Under the random effect model, by integrating the results of 9 studies [5, 7, 9, 11, 20–24], the final value of SMD was equal to 0.60. In other words, the circulating level of FABP-4 in people with CD is significantly higher than that of people without CD. These results are consistent with other studies [5, 7, 13, 21, 22, 25]. Besides, subjects with the highest number of stenotic vessels showed significantly higher serum FABP-4 levels compared with those with 1 vessel and those without CAD, suggesting the enlarging influence of FABP-4 levels as the extent of stenosis progressed [20].

The effect of gender on FABP-4 level has also been reported in past studies [9, 13, 22, 23]. Several studies have stated that the level of FABP-4 in women with CAD is higher than the level of FABP-4 in men with CAD [9, 13, 22]; however, a study reported that the circulating level of FABP-4 is only significant in women with CAD [9]. For this reason, in the present study, the effect of gender on FABP-4 level in patients with CD was investigated using metaregression analysis. The metaregression results showed that there is no significant relationship between gender and FABP-4 level. Probably, this gender difference in FABP-4 levels in the past studies is because of a higher amount of adipose tissue in women which is the main source of plasma FABP-4; further, sex hormones may also be involved in this difference [6]. The gender difference in the level of FABP-4 in past studies is partly linked to women's higher amount of body fat. Thus, to achieve more accurate results, the level of FABP-4 should be measured in proportion to other factors that are released from adipose tissue. To this end, a study measured the level of FABP-4 in proportion to adiponectin (FABP-4/adiponectin ratio). This index is less affected by age, gender, BMI, and other intervening factors [9]. On the other hand, the results of this study showed that the level of heterogeneity among the included studies is high (*I*^2^: 91.47%). One of the possible reasons for these heterogeneities can be drug use.

Race is another possible factor that affects this heterogeneity. A correlation between FABP-4 level and the development of coronary or carotid atherosclerosis and a correlation between FABP-4 level and overweight have been reported in Asian populations [7, 9]. Yet, one study showed that in the Caucasian population, there was no association between the serum level of FABP-4 and atherosclerosis [9, 26]. This difference can be partially attributed to the difference in the risk factors of coronary heart disease (CHD) in different populations [27, 28]. Among these CD risk factors, diabetes mellitus or insulin resistance is of higher importance in the Asian population than in the Caucasian population [22]. While a study showed that there is no relationship between circulating FABP-4 levels and insulin and homeostasis model assessment indices (HOMA-IR) in the Caucasian population [27], another study showed that there is a strong correlation between circulating FABP-4 levels and fasting insulin and homeostasis model assessment indices (HOMA-IR) in the Asian population [29]. For this reason, in the present study, we made a subgroup according to the countries where the initial studies were conducted. Metaregression analysis based on countries showed that this factor affects the heterogeneity of the current study. It seems that these results are due to the differences in the genetic structure of different populations and different FABP-4 genotypes, as well as its different polymorphisms in different populations.

Also, in the present study, in order to investigate the effective pathways and reasons for increasing the level of FABP-4, its relationship with BMI and lipid indices was measured. Although different results have been reported in this case in past studies [7, 10, 13, 30, 31], the results of the analyses showed that there is no significant relationship between FABP-4, BMI, and lipid indices including TG, HDL, LDL, and cholesterol. The absence of a clear association between circulating FABP-4 levels and BMI and lipid indices in this study can be attributed to the multifaceted nature of FABP-4 regulation and its role in CDs. FABP-4 expression is influenced by genetic, hormonal, and inflammatory factors, making it less reliant on BMI or lipid profiles alone. Additionally, the distribution of adipose tissue, genetic variability, insulin resistance, and inflammation all contribute to the complex interplay of FABP-4 in CDs. While FABP-4 may be a significant biomarker, its relationship with these parameters is intricate and not direct, underscoring the need for a comprehensive understanding of its role in disease development [11]. Being on medication and genetic difference can be attributed to the inconsistent results driven from past primary studies. Nonetheless, the results of the present study suggest that FABP-4 is not related to BMI and lipid indices.

In order to further investigate the factors affecting the evaluation, we also defined the subgroups of the type of primary studies and the type of kits, which showed that the type of studies and the type of kits have no effect on the heterogeneity of the studies. Also, the effect of the quality score on the heterogeneity of studies was investigated, and the results indicated that it had no effect on heterogeneity.

### 4.1. Limitation and Strong Points

Some factors effective in the effect of FABP-4 on CAD that could not be covered in our study, due to the lack of data, are considered significant limitations. The heterogeneity between the studies is one more. Therefore, we applied a random effect model to combine the primary results in this meta-analysis.

It did not account for medication usage, genetic factors, lifestyle variables, or diabetes-related factors that can influence FABP-4 levels. Meanwhile, previous studies have shown that treatment with atorvastatin can reduce the serum level of FABP-4 in patients with hyperlipidemia [20]. Additionally, the study primarily focused on FABP-4 without considering other adipokines or their combined effects. Longitudinal data, disease severity analysis, ethnic/racial considerations, and intervention studies were also missing. However, the study had some strong points, as well. A high number of studies were retrieved in the extensive search, and finally, 9 studies with a total sample size of 2327 were analyzed, which provides sufficient statistical power. The use of the complicate statistical model for the unification of SMD and the use of the trim-and-fill method for the adjustment of publication bias were the strong points of the present study. In addition, while the study highlighted an association between FABP-4 and CDs, future research should explore these unexamined factors to gain a more comprehensive understanding of FABP-4's role in cardiovascular health.

## 5. Conclusion

In conclusion, this meta-analysis examined circulating FABP-4 levels in CD patients, particularly CAD and atherosclerosis. The study, involving 9 studies with 2327 participants, found significantly higher FABP-4 levels in CD patients compared to controls, suggesting its potential as a biomarker. However, substantial study heterogeneity was noted, influenced by the country of study origin. Other factors like gender, BMI, lipid indices, study design, and kit type did not impact heterogeneity. No publication bias was observed. Further research should explore the clinical implications of elevated FABP-4 levels in CD, considering genetic and ethnic factors as well as other adipokines' role in these conditions.

## Figures and Tables

**Table 1:** Search strategy for Medline (MeSH (Medical Subject Headings)).

(1) Coronary Artery Disease [Text Word] OR Coronary Artery Disease [MeSH Terms]
(2) Cardiovascular Disease [Text Word] OR Cardiovascular Disease [MeSH Terms]
(3) Atherosclerosis [Text Word] OR Atherosclerosis [MeSH Terms]
(4) 1 OR 2 OR 3
(5) FABP-4 Protein, human [Text Word] OR FABP-4 Protein, human [MeSH Terms]
(6) Adipocyte lipid binding protein, human [Text Word] OR Adipocyte lipid binding protein, human [MeSH Terms]
(7) fatty acid-binding protein aP2, human [Text Word] OR fatty acid-binding protein aP2, human [MeSH Terms]
(8) 5 OR 6 OR 7
(9) Observational Studies [Text Word] OR Observational Studies [MeSH Terms]
(10) Cross-sectional Studies [Text Word] OR Cross-sectional Studies [MeSH Terms]
(11) Case-Control Studies [Text Word] OR Case-Control Studies [MeSH Terms]
(12) Cohort Studies [Text Word] OR Cohort Studies [MeSH Terms]
(13) 9 OR 10 OR 11 OR 12
(14) Serum [Text Word] OR Serum [MeSH Terms]
(15) Plasma [Text Word] OR Plasma [MeSH Terms]
(16) Blood [Text Word] OR Blood [MeSH Terms]
(17) 14 OR 15 OR 16
(18) 4 AND 8 AND 13 AND 17

**Table 2:** Baseline characteristics of the included primary studies that evaluated the FABP-4 level in patients with atherosclerosis or coronary artery disease in the present systematic review and meta-analysis.

Author	Year	Country	Method	Kit type	FABP-4 commercial kit type and company	Design	*N* case/*N* control	Age case (mean)	Age control (mean)	#FABP-4 (mean ± SD)		SMD (Se)	Secondary outcome (mean ± SD)		
										Case	Control		Subgroup	Case	Control
Rhee et al. [7]	2009	South Korea	ELISA	Sandwich ELISA	BioVendor	CS	135/99	60.7 ± 10	54.8 ± 11.9	11.7 ± 1.82	10.07 ± 2.01	0.86 (0.14)	BMI	25.7 ± 2.8	25.3 ± 2.9
													LDL	111.5 ± 32.4	112.9 ± 28.8
													HDL	47 ± 1.34	51.93 ± 1.22
													TG	148.41 ± 1.64	131.63 ± 1.64
													TC	186.79 ± 1.22	188.67 ± 1.22

Miyoshi et al. [20]	2010	Japan	ELISA	Sandwich ELISA	BioVendor	CC	125/120	67 ± 10	67 ± 6	28.8 ± 12	20.05 ± 8	0.85 (0.13)	BMI	24.3 ± 3.1	23.3 ± 2.7
													LDL	108.5 ± 24.9	106.8 ± 32.3
													HDL	47.2 ± 11.6	63 ± 17.4
													TG	145 ± 85.1	127.5 ± 69.8

Jin et al. [9]	2010	China	ELISA	Sandwich ELISA	BioVendor	CS	211/129	62.94 ± 9.97	60.99 ± 9.28	18 ± 6.8	17 ± 5	0.16 (0.11)	BMI	24.28 ± 3.32	23.93 ± 4.18
													LDL	101.7 ± 33.25	98.22 ± 32.9
													HDL	37.9 ± 9.28	41.38 ± 10.44
													TG	150.58 ± 113.37	121.35 ± 105.4
													TC	180.2 ± 117.56	167.83 ± 39.83

Holm et al. [21]	2011	Norway	ELISA	Sandwich ELISA	BioVendor	CS	28/31	65.3 ± 9.4	59 ± 6	29.1 ± 7.5	19 ± 4.3	1.65 (0.3)	BMI	28.9 ± 3.3	—
													LDL	96.67 ± 29	100.54 ± 23.2
													HDL	50.27 ± 15.46	54.14 ± 11.6
													TC	162.4 ± 35.19	166.28 ± 23.2

Doi et al. [22]	2011	Japan	ELISA	Sandwich ELISA	BioVendor	CC	211/211	66 ± 11	66 ± 10	21.75 ± 8.8	15.8 ± 6	0.79 (0.1)	BMI	24.9 ± 3.5	23.4 ± 2.9
													LDL	118.3 ± 27.4	104 ± 27.4
													HDL	43 ± 11	58 ± 12
													TG	136 ± 61.2	130.6 ± 35.8

Bao et al. [11]	2011	China	ELISA	Sandwich ELISA	BioVendor	CS	247/94	—	—	11.79 ± 7.52	11.43 ± 8.85	0.05 (0.12)	—	—	—

Hong et al. [23]	2011	China	ELISA	Solid phase sandwich ELISA	R&D Systems	CS	238/221	62.8 ± 8.5	61.4 ± 9.3	12.3 ± 5.45	10.94 ± 4.94	0.26 (0.09)	BMI	26.8 ± 2.7	23.8 ± 2.9
													LDL	104.4 ± 38.67	104.02 ± 30.55
													HDL	45.63 ± 13.14	46.79 ± 12.37
													TG	168.3 ± 90.35	173.6 ± 142.6
													TC	175.17 ± 41.76	177.88 ± 39.44

Kajiya et al. [5]	2013	Japan	ELISA	Sandwich ELISA	BioVendor	CC	115/50	69 ± 11	68 ± 6	22.4 ± 8.7	15.75 ± 5.3	0.85 (0.18)	BMI	25.7 ± 2.8	25.3 ± 2.9
													LDL	108.3 ± 28.1	105.8 ± 32.3
													HDL	43.2 ± 11.8	63 ± 17.4
													TG	165 ± 54.7	132.2 ± 64.8

Wu et al. [24]	2020	Taiwan	ELISA	Solid phase sandwich ELISA	R&D Systems	CC	22/40	66 ± 10	34 ± 7	5.24 ± 1.01	5.05 ± 1.03	0.19 (0.27)	BMI	25.5 ± 3.7	24.1 ± 3.6
													LDL	85 ± 22	105 ± 31
													HDL	47 ± 18	64 ± 18
													TG	144 ± 28	179 ± 29
													TC	118 ± 65	100 ± 55

Measurement unit of fatty acid-binding proteins (FABP)-4 was Ng/ml. SMD: standardized mean difference; Se: standard error; BMI: body mass index; LDL: low-density lipoprotein; HDL: high-density lipoprotein; TG: triglyceride; TC: total cholesterol.

## Data Availability

Data is available upon editor request.

## Supplementary Materials

Supplementary Materials Supplement Tables 1 and 2 show the results of the quality assessment of cross-sectional and case-control studies based on the Newcastle-Ottawa Quality Assessment Scale. Supplement Table 3 shows the results of the univariate metaregression analysis for the heterogeneity determinants. Also, Supplement Figure 1 shows the association among the FABP-4 with HDL (A) and LDL (B) by means of metaregression. Supplement Figure 2 indicates a funnel plot for publication bias assessment.

## References

[1] Stone N. J. (1996). The clinical and economic significance of atherosclerosis. *The American Journal of Medicine*.

[2] Khan M. A., Hashim M. J., Mustafa H. (2020). Global epidemiology of ischemic heart disease: results from the global burden of disease atudy. *Cureus*.

[3] Jahangir E., De Schutter A., Lavie C. J. (2014). The relationship between obesity and coronary artery disease. *Translational Research: the Journal of Laboratory and Clinical Medicine*.

[4] Rodríguez A., Becerril S., Hernández-Pardos A. W., Frühbeck G. (2020). Adipose tissue depot differences in adipokines and effects on skeletal and cardiac muscle. *Current Opinion in Pharmacology*.

[5] Kajiya M., Miyoshi T., Doi M. (2013). Serum adipocyte fatty acid-binding protein is independently associated with complex coronary lesions in patients with stable coronary artery disease. *Heart and Vessels*.

[6] Su X., Peng D. (2020). Adipokines as novel biomarkers of cardio-metabolic disorders. *Clinica Chimica Acta*.

[7] Rhee E. J., Lee W. Y., Park C. Y. (2009). The association of serum adipocyte fatty acid-binding protein with coronary artery disease in Korean adults. *European Journal of Endocrinology*.

[8] Saito N., Furuhashi M., Koyama M. (2021). Elevated circulating FABP4 concentration predicts cardiovascular death in a general population: a 12-year prospective study. *Scientific Reports*.

[9] Jin J., Peng D. Q., Yuan S. G. (2010). Serum adipocyte fatty acid binding proteins and adiponectin in patients with coronary artery disease: the significance of A-FABP/adiponectin ratio. *Clinica Chimica Acta*.

[10] Terra X., Quintero Y., Auguet T. (2011). FABP 4 is associated with inflammatory markers and metabolic syndrome in morbidly obese women. *European Journal of Endocrinology*.

[11] Bao Y., Lu Z., Zhou M. (2011). Serum levels of adipocyte fatty acid-binding protein are associated with the severity of coronary artery disease in Chinese women. *PLoS One*.

[12] Tso A. W., Xu A., Sham P. C. (2007). Serum adipocyte fatty acid binding protein as a new biomarker predicting the development of type 2 diabetes: a 10-year prospective study in a Chinese cohort. *Diabetes Care*.

[13] Furuhashi M., Tuncman G., Görgün C. Z. (2007). Treatment of diabetes and atherosclerosis by inhibiting fatty-acid-binding protein aP2. *Nature*.

[14] Page M. J., McKenzie J. E., Bossuyt P. M. (2021). The PRISMA 2020 statement: an updated guideline for reporting systematic reviews. *Systematic Reviews*.

[15] Haddaway N. R., Collins A. M., Coughlin D., Kirk S. (2015). The role of Google Scholar in evidence reviews and its applicability to grey literature searching. *PLoS One*.

[16] Modesti P., Reboldi G., Cappuccio F. (2016). Panethnic differences in blood pressure in Europe: a systematic review and meta-analysis. *PLoS One*.

[17] Wells G. A., Shea B., O’Connell D. (2000). *The Newcastle-Ottawa Scale (NOS) for assessing the quality of nonrandomised studies in meta-analyses*.

[18] Shaterian N., Pakzad R., Fekri S. D., Abdi F., Shaterian N., Shojaee M. (2022). Labor pain in different dilatations of the cervix and Apgar scores affected by aromatherapy: a systematic review and meta-analysis. *Reproductive Sciences*.

[19] Behesht Aeen F., Pakzad R., Goudarzi Rad M., Abdi F., Zaheri F., Mirzadeh N. (2021). Effect of prone position on respiratory parameters, intubation and death rate in COVID-19 patients: systematic review and meta-analysis. *Scientific Reports*.

[20] Miyoshi T., Onoue G., Hirohata A. (2010). Serum adipocyte fatty acid-binding protein is independently associated with coronary atherosclerotic burden measured by intravascular ultrasound. *Atherosclerosis*.

[21] Holm S., Ueland T., Dahl T. B. (2011). Fatty acid binding protein 4 is associated with carotid atherosclerosis and outcome in patients with acute ischemic stroke. *PLoS One*.

[22] Doi M., Miyoshi T., Hirohata S. (2011). Association of increased plasma adipocyte fatty acid-binding protein with coronary artery disease in non-elderly men. *Cardiovascular Diabetology*.

[23] Hong J., Gu W., Zhang Y. (2011). Different association of circulating levels of adipocyte and epidermal fatty acid-binding proteins with metabolic syndrome and coronary atherosclerosis in Chinese adults. *Atherosclerosis*.

[24] Wu Y. W., Chang T. T., Chang C. C., Chen J. W. (2020). Fatty-acid-binding protein 4 as a novel contributor to mononuclear cell activation and endothelial cell dysfunction in atherosclerosis. *International Journal of Molecular Sciences*.

[25] Rahnemaei F. A., Pakzad R., Amirian A., Pakzad I., Abdi F. (2022). Effect of gestational diabetes mellitus on lipid profile: a systematic review and meta-analysis. *Open Medicine*.

[26] Ginsberg H. N., MacCallum P. R. (2009). The obesity, metabolic syndrome, and type 2 diabetes mellitus pandemic: part I. Increased cardiovascular disease risk and the importance of atherogenic dyslipidemia in persons with the metabolic syndrome and type 2 diabetes mellitus. *Journal of the Cardiometabolic Syndrome*.

[27] Cabré A., Lázaro I., Girona J. (2007). Fatty acid binding protein 4 is increased in metabolic syndrome and with thiazolidinedione treatment in diabetic patients. *Atherosclerosis*.

[28] Yamamoto A., Richie G., Nakamura H. (2002). Risk factors for coronary heart disease in the Japanese--comparison of the background of patients with acute coronary syndrome in the ASPAC study with data obtained from the general population. Asia-Pacific collaboration on CHD risk factor intervention study. *Journal of Atherosclerosis and Thrombosis*.

[29] Woodward M., Zhang X., Barzi F. (2003). The effects of diabetes on the risks of major cardiovascular diseases and death in the Asia-Pacific region. *Diabetes Care*.

[30] Yeung D. C., Xu A., Cheung C. W. (2007). Serum adipocyte fatty acid-binding protein levels were independently associated with carotid atherosclerosis. *Arteriosclerosis, Thrombosis, and Vascular Biology*.

[31] Queipo-Ortuño M. I., Escoté X., Ceperuelo-Mallafré V. (2012). FABP4 dynamics in obesity: discrepancies in adipose tissue and liver expression regarding circulating plasma levels. *PLoS One*.

