# Supplementary material for: Circulating FABP-4 Levels in Patients with Atherosclerosis or Coronary Artery Disease: A Comprehensive Systematic Review and Meta-Analysis
Source: Cardiovasc Ther. 2023 Nov 17;2023:1092263. doi: 10.1155/2023/1092263 (PMC10673666; doi:10.1155/2023/1092263)
Supplement: Supplementary Materials — Supplement Tables 1 and 2 show the results of the quality assessment of cross-sectional and case-control studies based on the Newcastle-Ottawa Quality Assessment Scale. Supplement Table 3 shows the results of the univariate metaregression analysis for the heterogeneity determinants. Also, Supplement Figure 1 shows the association among the FABP-4 with HDL (A) and LDL (B) by means of metaregression. Supplement Figure 2 indicates a funnel plot for publication bias assessment. [file 1092263.f1.docx]

Supplement table 1: Quality Assessment of Quantitative Studies (Newcastle-Ottawa Quality Assessments Scale adapted for Cross Sectional Studies)

| Authors (Year) | Selection | | | | comparability | outcome | | Score |
| --- | --- | --- | --- | --- | --- | --- | --- | --- |
|  | 1: Representativeness of the sample | 2: Sample size | 3: non-respondents | 4: Ascertainment of the exposure (risk factor) | 1: The subjects in different outcome groups are comparable, based on the study design or analysis | 1: Assessment of the outcome | 2: Statistical test |  |
| Rhee et al. | --- | --- | * | ** | * | * | * | Satisfactory |
| holm et al. | * | --- | --- | ** | * | * | * | Satisfactory |
| Bao et al. | * | --- | * | ** | * | * | * | Good |
| Hong et al. | --- | --- | * | ** | * | * | * | Satisfactory |
| Jin et al. | --- | --- | * | ** | * | * | * | Satisfactory |

Supplement table 2: Quality Assessment of Quantitative Studies (Newcastle-Ottawa Quality Assessments Scale adapted for case-control Studies)

| Authors (Year) | Selection | | | | comparability | outcome | | | Score |
| --- | --- | --- | --- | --- | --- | --- | --- | --- | --- |
|  | 1: Representativeness of the exposed cohort | 2: Selection of the non-exposed cohort | 3: Ascertainment of exposure | 4: Demonstration that outcome of interest was not present at start of study | 1: Comparability of cohorts on the basis of the design or analysis controlled for confounders | 1: Assessment of outcome | 2: Was follow-up long enough for outcomes to occur | 3. Adequacy of follow-up of cohorts |  |
| Miyoshi et al. | * | * | --- | * | * | * | * | * | Good quality |
| Doi et al. | * | * | --- | * | * | * | * | * | Good quality |
| Kajiya et al. | * | * | --- | * | * | * | * | --- | Good quality |
| Wu et al. | * | --- | --- | --- | * | * | * | * | Fair quality |

Supplement Table 3: The univariate meta-regression analysis on the heterogeneity of the determinants in included studies for standardized mean difference of FABP-4.

| variables | coefficient | 95% CI | p-value |
| --- | --- | --- | --- |
| Kit type | -0.48 | -1.39 to 0.43 | 0.256 |
| Design | 0.15 | -0.67 to 0.98 | 0.674 |
| Country | 0.29 | 0.21 to 0.38 | <0.001* |
| Quality score | 0.06 | -0.61 to 0.74 | 0.826 |
| Gender | 0.05 | -0.51 to 0.62 | 0.791 |
| #HDL | -0.09 | -0.47 to 0.29 | 0.587 |
| #BMI | -0.32 | -1.37 to 0.74 | 0.472 |
| #LDL | 0.26 | -1.15 to 1.66 | 0.670 |
| #TG | 0.04 | -0.05 to 0.13 | 0.340 |
| #TC | -0.31 | -1.75 to 1.13 | 0.541 |
| *:significant at 0.05  Kit type code: Sandwich ELISA=1; Solid Phase Sandwich ELISA=2  Design: cross sectional=1; case control=2  Gender: Male=1; Female=2  Country: China=1; Taiwan=2; Japan=3; South Korea=4; Norway=5  #: standardized mean difference | | | |


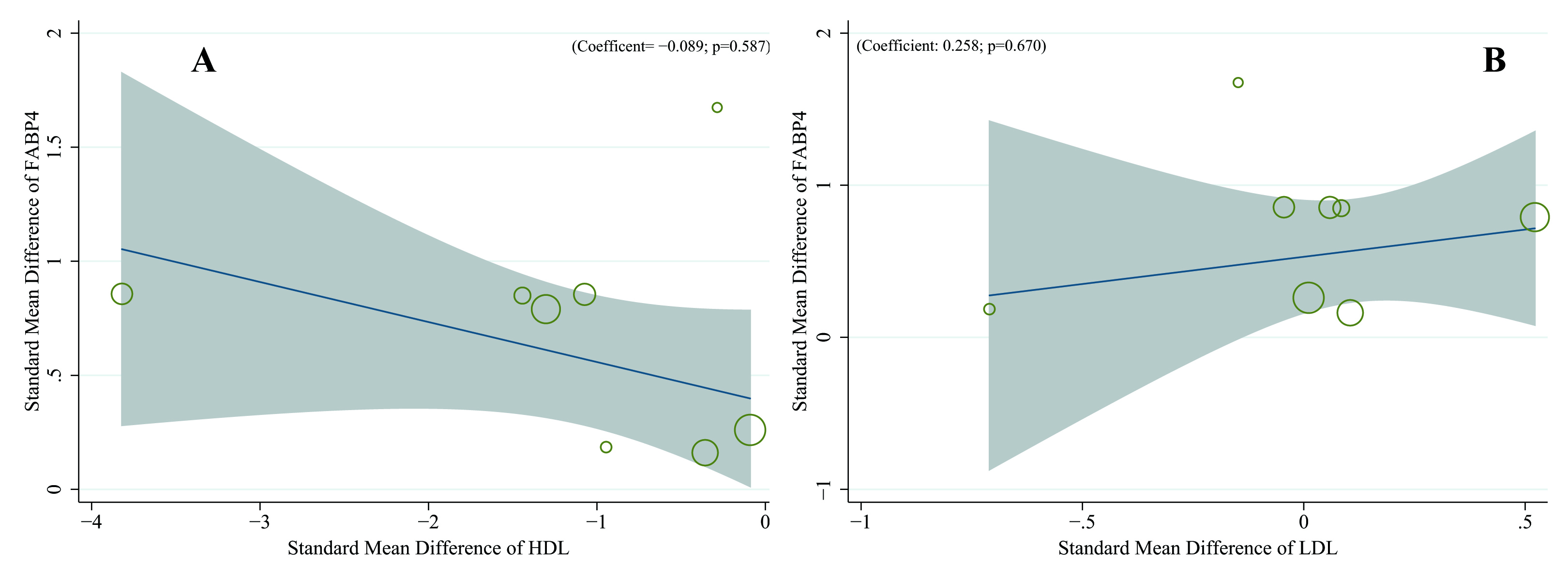


Supplement Figure 1. Association among the standardized mean difference of FABP-4 standardized mean difference of HDL (A) and LDL (B) by means of meta-regression. The size of circles indicates the precision of each study. There is no significant association between FABP-4 with LDL and HDL.


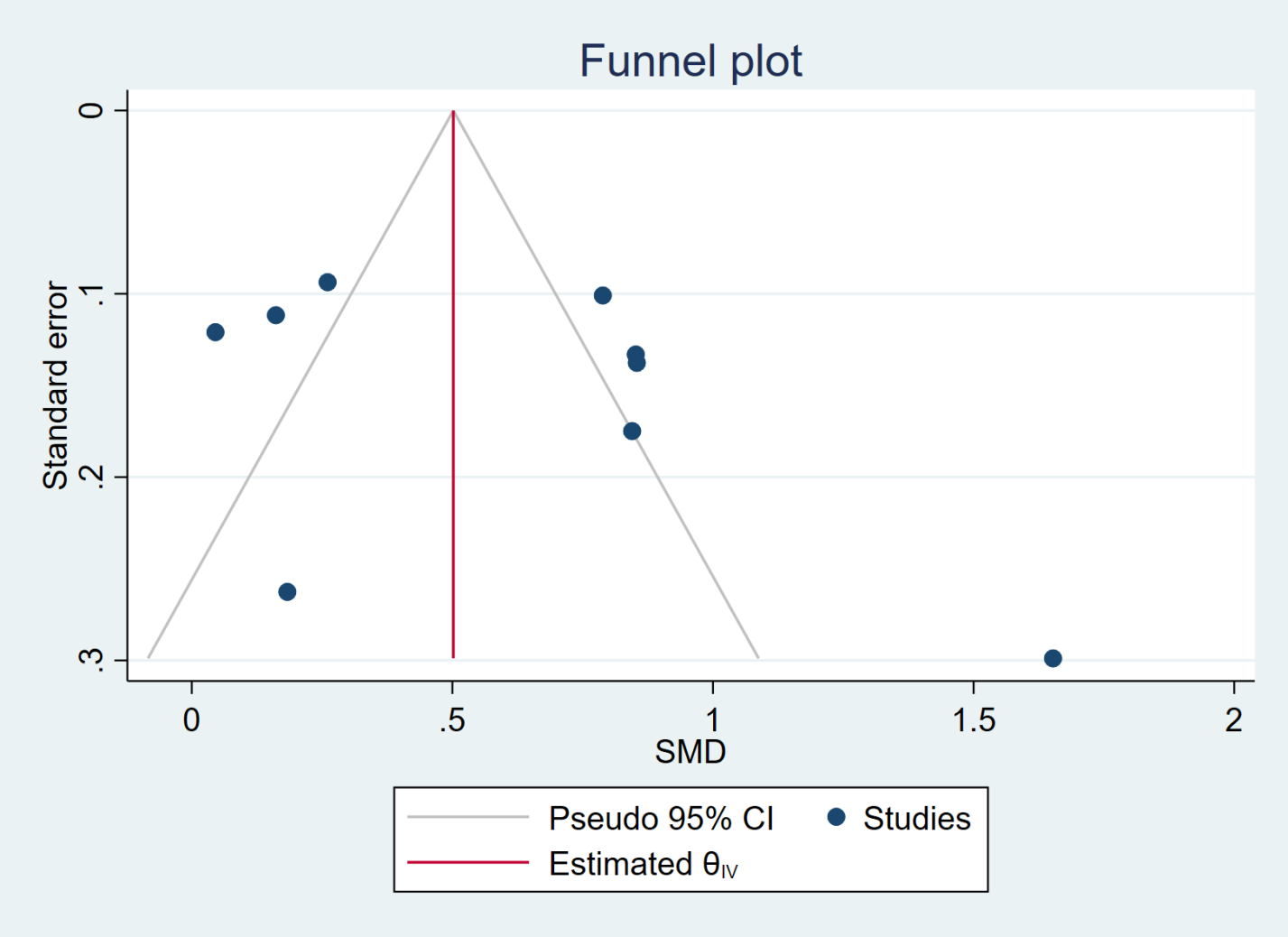


Supplement Figure 2. Funnel plot for publication bias assessment. The horizontal and vertical axes represent the standardized mean difference and the standard error, respectively.
